# Fishes of the Cusiana River (Meta River basin, Colombia), with an identification key to its species

**DOI:** 10.3897/zookeys.733.20159

**Published:** 2018-01-26

**Authors:** Alexander Urbano-Bonilla, Gustavo A. Ballen, Guido A. Herrera-R, Jhon Zamudio, Edgar E. Herrera-Collazos, Carlos DoNascimiento, Saúl Prada-Pedreros, Javier A. Maldonado-Ocampo

**Affiliations:** 1 Laboratorio de Ictiología, Unidad de Ecología y Sistemática (UNESIS), Departamento de Biología, Facultad de Ciencias, Pontificia Universidad Javeriana, Carrera 7 N° 43-82, Bogotá, D.C., Colombia; 2 Museu de Zoologia da Universidade de São Paulo, Caixa Postal 42494, 04218-970 São Paulo, SP, Brazil; 3 Université Paul Sabatier, 118 Route de Narbonne, 31062, Toulouse, France; 4 Grupo de Investigaciones territoriales para el uso y conservación de la Biodiversidad, Fundación Reserva Natural La Palmita - Centro de Investigación, Carrera 4 N° 58-59, Oficina 301, Bogotá, D.C., Colombia; 5 Colecciones del Instituto de Investigación de Recursos Biológicos Alexander von Humboldt, Claustro de San Agustín, Carrera 8 N° 15-08. Villa de Leyva, Colombia

**Keywords:** Andean Orinoquia, Casanare, conservation, diversity, Llanos, Piedmont, species richness, Diversidad, Casanare, Conservación, Llanos, Orinoquia Andina, Piedemonte, Riqueza de especies

## Abstract

The Cusiana River sub-basin has been identified as a priority conservation area in the Orinoco region in Colombia due to its high species diversity. This study presents an updated checklist and identification key for fishes of the Cusiana River sub-basin. The checklist was assembled through direct examination of specimens deposited in the main Colombian ichthyological collections. A total of 2020 lots from 167 different localities from the Cusiana River sub-basin were examined and ranged from 153 to 2970 m in elevation. The highest number of records were from the piedmont region (1091, 54.0 %), followed by the Llanos (878, 43.5 %) and Andean (51, 2.5 %). 241 species distributed in 9 orders, 40 families, and 158 genera were found. The fish species richness observed (241), represents 77.7 % of the 314 estimated species (95 % CI=276.1–394.8). The use of databases to develop lists of fish species is not entirely reliable; therefore taxonomic verification of specimens in collections is essential. The results will facilitate comparisons with other sub-basins of the Orinoquia, which are not categorized as areas of importance for conservation in Colombia.

## Abstract (es)

La sub-cuenca del rio Cusiana ha sido designada como una de las áreas prioritarias para la conservación en la región del Orinoco en Colombia debido a su alta diversidad de especies. Este estudio presenta una lista actualizada y una clave de identificación para los peces del área. Para ello se revisaron los especímenes depositados en las principales colecciones ictiológicas colombianas. Se examinaron un total de 2020 lotes de 167 localidades diferentes de la sub-cuenca del río Cusiana, que oscilaron entre 153 y 2970 m en altitud. El mayor número de registros corresponde a la región del piedemonte (1091, 54.0%), seguida por los Llanos (878, 43.5%) y Andina (51, 2.5%). Encontramos 241 especies distribuidas en 9 órdenes, 40 familias y 158 géneros. La riqueza de especies de peces observada (241), representan el 77.7% de las 314 especies estimadas (IC 95% = 276.1-394.8). El uso de bases de datos para elaborar listas de especies de peces no es del todo fiable, por lo tanto, es esencial la verificación taxonómica de los especímenes en las colecciones. Estos resultados permitirán evaluar otras sub-cuencas de la Orinoquia, las cuales no están categorizadas como áreas de importancia para la conservación en Colombia.

## Introduction

The Orinoco River, with an estimated richness of 1002 species of freshwater fishes, is the second most diverse drainage in the Neotropical region (Reis et al. 2016). Nonetheless, the basin has been exposed to increasing threats due to human activities that place the enormous fish diversity at risk (Barletta et al. 2010, Rybicki and Hanski 2013, Lasso et al. 2016). The systems draining the Andean region (western tributaries of the Orinoco) are considered the most threatened at basin scale (Rodríguez et al. 2007, Machado-Allison et al. 2010, Lasso et al. 2016). The rivers originating in the Andes are heavily exposed to threats like habitat fragmentation, contamination, deforestation, the introduction of non-native species and mining (Machado-Allison et al. 2010, Anderson and Maldonado-Ocampo 2011, Lasso et al. 2016). Additionally, large gaps regarding the basic knowledge of fish diversity of the Andean sub-basins are persistent, especially in Colombia (Maldonado-Ocampo et al. 2008, Machado-Allison et al. 2010, Lasso et al. 2016). Filling those gaps are essential to guide adequate conservation efforts for the freshwater ecosystems and therefore face the threats above mentioned.

The Meta River basin, with headwaters on the Eastern Cordillera of Colombia, is one of the major tributaries of the Orinoco River (Usma-Oviedo et al. 2016). Studies on its fish diversity (e.g., Urbano-Bonilla et al. 2009, 2014, Villa-Navarro et al. 2011, Maldonado-Ocampo et al. 2013, Urbano-Bonilla and Maldonado-Ocampo 2013), and recent efforts (Zamudio et al. 2008, Urbano-Bonilla et al. 2016) have advanced our understanding of the ecology of some species. The Cusiana River sub-basin is one of the best-known Andean tributaries of the Meta River basin; the first inventories of its fish diversity dated back to the 90’s with the establishment of oil companies in the area. The Cusiana River sub-basin has been considered as a conservation priority area for the Orinoco biodiversity due to its high diversity in several groups (Lasso et al. 2010, Trujillo et al. 2011), including fishes (Villa-Navarro et al. 2011).

Here an updated checklist and an identification key are presented for the fishes of the Cusiana River sub-basin. We hope our results may establish a guideline that can be replicated in other basins of the Orinoco drainage.

## Materials and methods

**Figure 1.**
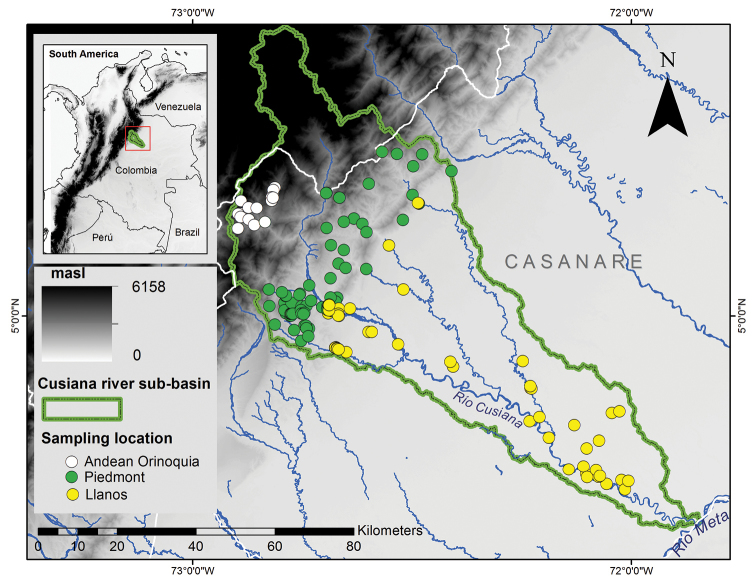
Collection localities in the Cusiana River sub-basin.

The Cusiana River sub-basin has an extension of 7324 km² and 271 km in length, originating at 3800 m asl on the eastern slope of the Eastern Cordillera in the Quebradas La Iglesia, Melgarejo, and Las Cañas, Boyacá Department (5°35'N, 72°47'W), and empties at 150 m asl in the Meta river, Casanare Department (4°31'N, 71°51'W) (IGAC 1999) (Fig. 1). The Cusiana River sub-basin was divided by altitudinal limits in three distinctive regions based on Abell et al. (2008): Llanos (139–300 m asl), Piedmont (300–1235 m asl) and High Andes (1235–3000 m asl).

The checklist was assembled by examining specimens deposited in Colombian ichthyological collections. Acronyms used in the text follow Sabaj-Pérez (2016) except uncatalogued material housed at Fundación Universitaria del Trópico Americano (UNITROPICO). The taxonomic list follows the classification system proposed by Reis et al. (2003) with recent modifications proposed by Oliveira et al. (2011) for characiform families, Betancur-R et al. (2016) at high-level groups for osteichthyans in general, and Thomaz et al. (2015) for genera of the Stevardiinae. Valid species names were confirmed through queries on the Catalog of Fishes of the California Academy of Sciences (Eschmeyer et al. 2017). Species were categorized as endemic (DoNascimiento et al. 2017), threatened (Mojica et al. 2012), migratory (Usma-Oviedo et al. 2013), and species subject of conservation (González et al. 2015).

Species richness interpolation and extrapolation was calculated following Chao et al. (2014) and using the package iNEXT 2.0.12 (Hsieh et al. 2016) for R v.3.4.0 (R Core Development Team 2017). The number of localities were obtained per Orinoco basin from the data set of the “Catalogue of the Freshwater Fishes of Colombia” (DoNascimiento et al. 2017).

To construct the key (for order, families and species), original descriptions of species, taxonomic revisions, and direct examination of specimens were used. Finally, in order to share the information produced herein, the dataset was uploaded to SiB Colombia’s data portal (GBIF Colombia Node) in accordance with their protocols for species lists. For the latter, the complete dataset was structured and standardized to comply with the international biodiversity standard: Darwin Core standard (Wieczorek et al. 2012). After mounting the dataset on a Darwin Core spreadsheet template, it was uploaded to SiB Colombia’s Integrated Publishing Tool for international visualization in their data portal. A DOI was provided by SiB Colombia for the shared dataset available at http://doi.org/10.15472/er3svl, all the results, discussion and quantities herein cited follow the version 1.8 of the published dataset.

## Results

**Figure 2.**
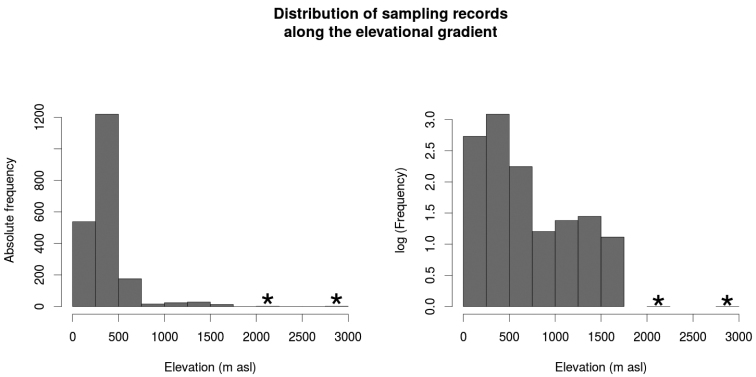
Distribution of sampling records along the Elevational gradient in the Cusiana River sub-basin. Asterisks indicate categories with the lowest sampling along the elevational gradient.

**Figure 3.**
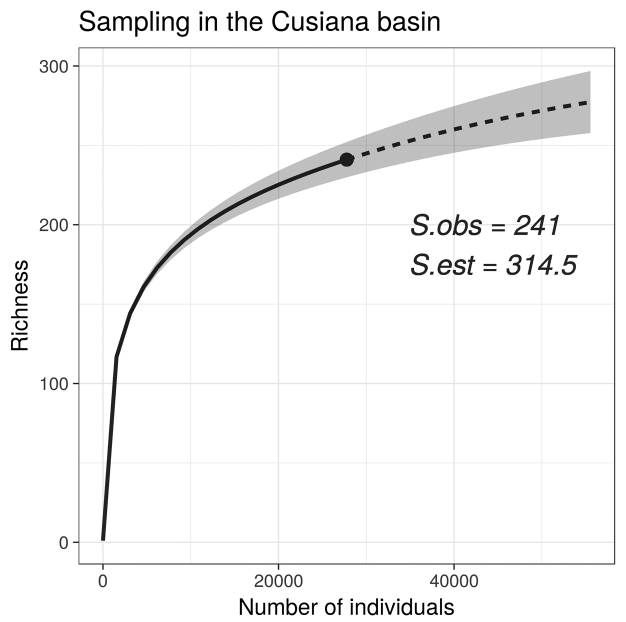
Species accumulation curve in the Cusiana River sub-basin. Abbreviations: S.obs = observed richness, S.est = estimated richness. Continuous line represents interpolation, and dashed line represents extrapolation.

In total, 2020 lots from 167 different localities from the Cusiana River sub-basin ranging from 152 to 2970 m asl were examined. Most of the records were found in the piedmont (1091, 54.0%), followed by the llanos (878, 43.5%) and Andean Orinoquia (51, 2.5%), suggesting sampling bias in elevation for this drainage, being inversely-proportional to elevation (Fig. 2). The number of localities in Cusiana River sub-basin represents the quantile 0.83 among the tributaries of the Orinoco drainage in Colombia (Table 1). 241 species were found distributed in nine orders, 40 families, and 158 genera. The order Characiformes showed the highest richness with 106 species, followed by Siluriformes (89), Gymnotiformes (20), and Cichliformes (15). The remaining orders were represented by one to three species. The most speciose families were the Characidae (54), Loricariidae (30), Cichlidae (15), Heptapteridae (15), Pimelodidae (11), and Curimatidae (9), while the 34 remaining families were represented by 1 - 8 species. Extrapolation suggests that the expected richness for the Cusiana River sub-basin is roughly 314 species (95% CI = 276.1–394.8) (Fig. 3); with the observed richness corresponding to around 77.7% the expected richness.

**Table 1.** Number of sampling sites per basin.

Basin	Number of sampling locations
Guamal-Humadea	213
Guacavía-Guatiquía-Humea	174
Cusiana	167
Ariari	74
Upía	61
Guayuriba	57
Cravo-Norte	56
Pauto	56
Cravo-Sur	53
Arauca	43
Guayabero	35
Tua	29

Concerning threatened species, five are currently categorized as Vulnerable (*Potamotrygon motoro*, *Brachyplatystoma vaillantii*, *Pseudoplatystoma metaense*, *P. orinocoense*, and *Zungaro zungaro*), and two as Nearly Threatened (*Potamotrygon orbignyi* and *Sorubim lima*). There are 34 species endemic to the Orinoco drainage, 20 are migratory, and 8 are subjects of conservation. A total of 50 species are new records for the Cusiana River sub-basin, while *Cetopsorhamdia shermani* and *Rhamdia muelleri* are also new records for Colombia. Some species from the genera *Andinoacara*, *Astroblepus*, *Ceratobranchia*, *Cetopsorhamdia*, *Chaetostoma*, *Characidium*, *Corydoras*, *Creagrutus*, *Curimatopsis*, *Hypostomus*, *Imparfinis*, *Microglanis*, *Parodon*, *Parotocinclus*, *Pimelodella*, *Poecilia*, *Spatuloricaria*, and *Trichomycterus*, require further revision by specialists.

### Key to orders, families, and species of fishes of the Cusiana River sub-basin

**Table:** 

1	Five pairs of gills in ventral position	**MYLIOBATIFORMES**: **Potamotrygonidae** (2 species)
–	Two lateral gill openings, or just one gill opening under head	**2**
2	Eyes located on the same side of body	**PLEURONECTIFORMES**: **Achiridae** (2 species)
–	Eyes located on opposite sides of body	**3**
3	Dorsal fin absent	**4**
–	Dorsal fin present	**5**
4	Anal fin absent; one ventral gill opening	**SYNBRANCHIFORMES**: **Synbranchidae: *Synbranchus marmoratus***
–	Anal fin present, always long, two lateral gill openings	**GYMNOTIFORMES** (20 species)
5	Body naked or covered with bony plates; barbels present near the mouth	**SILURIFORMES** (90 species)
–	Body totally or partially covered with scales; barbels absent	**6**
6	Dorsal and anal fins with two or more spines; scales ctenoid	**7**
–	Dorsal and anal fins without spines; scales cycloid (ctenoid in some groups)	**8**
7	Lateral line interrupted	**CICHLIFORMES** (15 species)
–	Lateral line continuous	**ACANTHURIFORMES**: **Sciaenidae** (2 species)
8	Mouth superior and somewhat protractile; adipose fin absent	**CYPRINODONTIFORMES** (3 species)
–	Mouth usually in terminal position, never protractile; adipose fin usually present	**CHARACIFORMES** (106 species)
**MYLIOBATIFORMES**		
**Potamotrygonidae**		
1	Dorsum brown with yellow or orange regularly distributed ocelli, ocelli rarely fused; teeth of both jaws in adult males with cusps on the central axis, teeth in females flattened in all rows	***Potamotrygon motoro***
–	Dorsum light brown with black or dark brown spots forming reticulate hexagonal lattices, mainly on the interorbital region; teeth small with triangular slightly monocuspid crowns in males and trapezoid tricuspids in females	***Potamotrygon orbignyi***
**PLEURONECTIFORMES**		
**Achiridae**		
1	Pectoral fins present; gill openings wide and extending ventrally on both sides of head	***Hypoclinemus mentalis***
–	Pectoral fins absent; gill openings limited to a narrow slit and never connected ventrally to both sides of head	***Apionichthys sauli***
**GYMNOTIFORMES**		
1	Caudal fin and dorsal filament present	**Apteronotidae**
–	Caudal fin and dorsal filament absent	**2**
2	Mouth in upper position, body cylindrical	**Gymnotidae**
–	Mouth terminal or subterminal, body compressed	**3**
3	Teeth present; nares tubular	**Sternopygidae**
–	Teeth absent; anterior nares not tubular	**4**
4	Absence of mental accessory electric organ	**Hypopomidae**: ***Brachyhypopomus brevirostris***
–	Presence of mental accessory electric organ	**Rhamphichthyidae**
**Apteronotidae**		
1	Lower jaw with a distinct V-shaped median groove accommodating the pointed decurved upper jaw	***Adontosternarchus devenanzii***
–	Lower jaw without a V-shaped median groove	**2**
2	Snout tubular	**3**
–	Snout obtuse or elongate but not tubular	**5**
3	Absence of teeth on upper jaw	***Platyurosternarchus macrostoma***
–	Presence of teeth on upper jaw	**4**
4	Total anal-fin rays 212–242	***Sternarchorhynchus oxyrhynchus***
–	Total anal-fin rays 193–210	***Sternarchorhynchus roseni***
5	Mid-dorsal pale stripe absent	***Compsaraia compsa***
–	Mid-dorsal pale stripe present	**6**
6	Presence of two clear bands surrounding caudal peduncle	***Apteronotus albifrons***
–	Presence of a single clear band surrounding base of caudal peduncle	**7**
7	More than 10 scales above lateral line	***Apteronotus galvisi***
–	10 or fewer scales above lateral line	***Apteronotus bonapartii***
**Gymnotidae**		
1	Without a particular color pattern on body; anal fin confluent with tail	***Electrophorus electricus***
–	Body color pattern formed by dark oblique bands alternating with pale bands; anal fin not confluent with tail	**2**
2	Obliquely-oriented dark bands or band pairs with straight or high-contrast margins, 23 dark bands (usually more than 30)	***Gymnotus cataniapo***
–	Obliquely-oriented dark bands or band pairs with irregular and wavy margins 23	***Gymnotus carapo septentrionalis***
**Sternopygidae**		
1	Orbital margin free; all anal-fin rays simple; background color variable from uniformly gray to black; humeral black blotch present, rarely diffused; white narrow band under the lateral line, from the midbody to end of anal fin	***Sternopygus macrurus***
–	Orbital margin continuous; background color variable from translucent to white; humeral blotch absent	**2**
2	Anal fin either completely black or with a black margin; no horizontal dark stripes on body; scales above lateral line 15–18	***Eigenmannia limbata***
–	Anal fin hyaline; 2 or 3 horizontal dark stripes on body; scales above lateral line 8–11	***Eigenmannia* sp.**
**Rhamphichthyidae**		
1	Anterior nares inside upper lip	**2**
–	Anterior nares outside upper lip	**3**
2	Absence of sixth infraorbital bone	***Hypopygus lepturus***
–	Presence of sixth infraorbital bone, as a narrow tube, positioned vertically, parallel to posterior border of eye	***Hypopygus neblinae***
3	Number of pectoral-fin rays fewer than 16	***Gymnorhamphichthys hypostomus***
–	Number of pectoral-fin rays more than 16	**4**
4	Anal fin usually clear or hyaline	***Rhamphichthys apurensis***
–	Anal fin usually dark with a terminal dark band	***Rhamphichthys rostratus***
**CYPRINODONTIFORMES**		
1	Pectoral fin with 1–2 unbranched rays	**Poeciliidae: *Poecilia* sp.**
–	Pectoral fin with all rays branched	**Cynolebiidae**
**Cynolebiidae**		
1	Scales on the ventral surface of the head	***Anablepsoides* sp.**
–	Scales absent on the ventral surface of the head	***Rachovia maculipinnis***
**SILURIFORMES**		
1	Mental barbels absent; ventral mouth in the form of a sucking disk with wide lower lip	**2**
–	Mental barbels present; terminal mouth	**3**
2	Body naked	**Astroblepidae**
–	Body covered with bony plates	**Loricariidae**
3	Body depressed; skin covered with tubercles and completely keratinized	**Aspredinidae**
–	Body not depressed; skin without tubercles	**4**
4	Body covered with bony plates	**5**
–	Body not covered with bony plates	**6**
5	Sides of body covered with two rows of bony plates	**Callichthyidae**
–	Sides of body with a mid-lateral row of dermal plates armed with a lateral thorn	**Doradidae**
6	Opercle and interopercle with odontodes	**Trichomycteridae**
–	Opercle and interopercle without odontodes	**7**
7	Lateral-line system branched on head	**Pimelodidae**
–	Lateral-line system simple, not branched on head	**8**
8	Suborbital sulcus present	**Auchenipteridae**
–	Suborbital sulcus absent	**9**
9	Adipose fin absent	**Cetopsidae**
–	Adipose fin present	**10**
10	Eyes set on anteriormost half of head, thick skin	**Pseudopimelodidae**
–	Eyes set on posteriormost half of head, thin skin	**Heptapteridae**
**Astroblepidae**		
1	Adipose fin present	***Astroblepus* sp.**
–	Adipose fin absent	***Astroblepus mariae***
**Loricariidae**		
1	Tail strongly depressed	**2**
–	Tail oval in cross-section	**12**
2	Dorsal-fin insertion in front of the anal-fin insertion	**3**
–	Dorsal-fin insertion anterior to the anal-fin insertion	**5**
3	Three rows of abdominal plates	***Farlowella mariaelenae***
–	Two rows of abdominal plates	**4**
4	Breeding odontodes on preorbital ridge present	***Farlowella acus***
–	Breeding odontodes on preorbital ridge absent	***Farlowella vittata***
5	Teeth villiform	**6**
–	Teeth spoon-shaped, elongate, comb-shaped or even absent but never villiform	**7**
6	Pectoral-fin rays i,7	***Lamontichthys llanero***
–	Pectoral-fin rays i,6	***Sturisoma tenuirostre***
7	Premaxillary teeth larger than dentary teeth	***Loricaria cataphracta***
–	Premaxillary teeth equal to or smaller than dentary teeth	**8**
8	Snout round in outline; upper lip with long filaments covering mouth opening	***Dentectus barbarmatus***
–	Snout acute to ovoid in outline; filaments covering mouth opening absent	**9**
9	Dentary teeth larger than premaxillary teeth	***Rineloricaria eigenmanni***
–	Dentary teeth smaller than or equal to premaxillary teeth	**10**
10	A pair of digitiform papillae on rictal region of mouth and an additional pair on mouth roof	***Spatuloricaria* sp.**
–	Digitiform papillae on rictal region of mouth but absent on its mouth roof	**11**
11	Anterior margin of abdominal plate cover oval in outline; fringes on upper lip darkly pigmented	***Loricariichthys brunneus***
–	Anterior margin of abdominal plate cover irregular in outline; fringes on upper lip unpigmented	***Limatulichthys griseus***
12	Snout naked, not covered with plates	**13**
–	Snout covered with plates	**17**
13	Fleshy tentacles on snout present; three series of lateral plate series on caudal peduncle	***Ancistrus triradiatus***
–	Fleshy tentacles on snout absent; five series of lateral plate series on caudal peduncle	**14**
14	Cheek odontodes straight; fleshy excrescence on parieto-supraoccipital absent	***Chaetostoma dorsale***
–	Cheek odontodes curved; fleshy excrescence on parieto-supraoccipital present	**15**
15	No dark spots on any fin but dark stripes present along rays in all fins; enlarged second unbranched ray in anal fin bearing two fleshy ridges in mature males	***Chaetostoma formosae***
–	Dark spots on fins; anal fin never bearing paired fleshy ridges in mature males	**16**
16	Ventral portion of body posterior to pelvic-fin insertion light, never covered with abundant dark spots; pectoral-fin spine with dark spots	***Chaetostoma* sp.**
–	Body uniformly spotted; pectoral-fin spine without spots	***Chaetostoma joropo***
17	Posterior serrae on pectoral-fin spine present	***Hypoptopoma machadoi***
–	Posterior serrae on pectoral-fin spine absent	**18**
18	Spoon-shaped teeth	**19**
–	Villiform teeth	**21**
19	Cheek odontodes absent	***Hypostomus plecostomoides***
–	Cheek odontodes present and erectile	**20**
20	Dark background with yellow or white vertical and irregular bands; small adult size	***Panaqolus maccus***
–	Color pattern consisting of horizontal stripes; large adult size	***Panaque nigrolineatus***
21	Eyes visible in ventral view; adipose fin absent	**22**
–	Eyes not visible in ventral view; adipose fin present	**23**
22	Twenty-six or more premaxillary teeth, 21 or more mandibular teeth; teeth slender, tightly spaced along the jaw rami, spacing between teeth equal to or greater than the tooth shaft width, tooth cusps small	***Otocinclus huaorani***
–	Fewer than 25 premaxillary teeth, 20 mandibular teeth; teeth robust, spacing between teeth equal to or greater than tooth shaft width, tooth cusps robust	***Otocinclus vittatus***
23	Coracoid bearing odontodes in ventral surface	***Parotocinclus* sp.**
–	Coracoid covered with skin and not bearing odontodes	**24**
24	Setiform odontodes on sides of head; three series of lateral plates on caudal peduncle	***Lasiancistrus tentaculatus***
–	No setiform odontodes on sides of head; five series of lateral plates on caudal peduncle	**25**
25	Hypertrophied cheek odontodes surpassing base of pectoral fin; pectoral-fin spine reaching or surpassing tip of pelvic-fin leading ray	***Dolichancistrus fuesslii***
–	No hypertrophied cheek odontodes; pectoral-fin spine not reaching tip of pelvic-fin leading ray	**26**
26	Multiple buccal papillae	***Aphanotorulus ammophilus***
–	Single medial buccal papilla	**27**
27	More than 11 branched dorsal-fin rays	***Pterygoplichthys multiradiatus***
–	Less than 11 branched dorsal-fin rays	**28**
28	Lower caudal-fin lobe dark and upper lobe light, no spots on caudal-fin; caudal peduncle elongated	***Aphanotorulus emarginatus***
–	Spots on caudal fin, no distinct dark background on lower caudal-fin lobe; caudal peduncle not elongated	**29**
29	Dark blotches closely-set on dorsal fin	***Hypostomus niceforoi***
–	Dark spots arranged in two longitudinal series on interradial membranes, no blotches on dorsal fin	***Hypostomus argus*** (please note that these differences in coloration are preliminary observations based on limited samples along with type specimens, further and more extensive sampling will be required in order to test such differences between *Hypostomus argus* and *H. niceforoi* in the Orinoco basin of Colombia; G.A. Ballen & S. Reinales, in prep.)
**Aspredinidae**		
1	Dorsal, ventral and lateral series of bony plates present on body; mouth inferior; hard pectoral spine, without serrae on the anterior margin	***Hoplomyzon sexpapilostoma***
–	No obvious bony plates on body; mouth terminal; hard pectoral spine serrate on anterior and posterior margins	***Bunocephalus aloikae***
**Callichthyidae**		
1	Snout depressed; maxillary barbel long, usually extending beyond gill opening	**2**
–	Snout compressed; maxillary barbel short, usually not extending beyond eye	**3**
2	Caudal fin forked, without conspicuous dark bands; dorsal fin spine about half the length of the first branched rays; six branched anal-fin rays	***Hoplosternum littorale***
–	Caudal fin truncated, with dark transverse band and dark distal margin; dorsal-fin spine long (52–64% dorsal-fin base length); four or five branched anal-fin rays	***Megalechis picta***
3	Dorsal fin with a dark blotch	***Corydoras* sp.**
–	Dorsal fin without dark blotches	**4**
4	A dark oblique stripe on body runs across dorsolateral body scutes to caudal-fin base. No vertical bars on caudal peduncle or on caudal fin	***Corydoras axelrodi***
–	A large dark blotch on the sides extending from slightly in front of dorsal fin to nearly adipose fin, tapering off posteriorly. A vertical bar in the caudal peduncle. Five to seven vertical bars on the caudal fin	***Corydoras septentrionalis***
**Doradidae**		
1	Maxillary barbel branched	***Leptodoras nelsoni***
–	Maxillary barbel simple, not ramified	**2**
2	Anterior and posterior dorsal-fin serrae absent	***Amblydoras affinis***
–	Anterior, posterior, or both dorsal-fin serrae present	**3**
3	Procurrent caudal-fin rays modified in fulcra; dark background with clear longitudinal stripe	***Platydoras armatulus***
–	Procurrent caudal-fin rays not modified in fulcra; color pattern without a clear longitudinal stripe	**4**
4	Thick lips, premaxillary and dentary teeth absent	***Oxydoras sifontesi***
–	Thin lips, premaxillary and dentary teeth present	**5**
5	Small body, caudal truncate	***Amblydoras bolivarensis***
–	Large body, caudal emarginate with two distinct lobes	***Pterodoras rivasi***
**Trichomycteridae**		
1	Nasal barbel present (associated with anterior nostril), mouth subterminal	**2**
–	Nasal barbel absent, mouth ventral	**4**
2	Sides of body with a dark band or row of spots from just above the gill-opening to the base of the upper caudal-fin rays	***Trichomycterus dorsostriatus***
–	Sides of body uniformly pigmented	**3**
3	Eight branched pectoral-fin rays (rarely nine rays); a single median epiphyseal pore (rarely two asymmetrical pores)	***Trichomycterus* sp.**
–	Seven branched pectoral-fin rays (rarely six rays); a pair of symmetrical epiphyseal pores	***Trichomycterus migrans***
4	Lower jaw rami not articulated medially, only joined by soft tissue	***Vandellia beccarii***
–	Lower jaw rami medially articulated	**4**
4	Caudal peduncle with numerous procurrent caudal-fin rays, embedded in wide dorsal and ventral membranes continuous with the caudal fin, caudal fin rounded or truncated	***Ochmacanthus alternus***
–	Caudal peduncle with few procurrent caudal-fin rays, restricted to the origin of the caudal fin, and lacking wide dorsal and ventral membranes continuous with the caudal fin, caudal fin emarginated	***Schultzichthys bondi***
**Cetopsidae**		
1	First ray of pectoral and dorsal fins spinous, lateral line extending to over the posterior portion of the base of the anal fin but falling short of the caudal peduncle	***Cetopsidium pemon***
–	First ray of pectoral and dorsal fins not spinous, lateral line either shorter or extending posteriorly at least onto the posterior portion of the caudal peduncle	***Cetopsis orinoco***
**Heptapteridae**		
1	Eye margin free, first dorsal and pectoral-fin rays with a pungent spine	**2**
–	Eye margin not free, first dorsal and pectoral-fin rays soft and segmented (at least their distal part)	**6**
2	Occipital process in contact with the nuchal plate, posterior cranial fontanelle open	**3**
–	Occipital process not reaching posteriorly the nuchal plate, posterior cranial fontanelle closed or reduced	**5**
3	Adipose-fin equals to one third of SL or shorter	***Pimelodella metae***
–	Adipose-fin longer than one third of SL	**4**
4	Upper caudal-fin lobe narrower and shorter than the lower caudal-fin lobe	***Pimelodella cristata***
–	Upper caudal-fin lobe about same width than lower lobe and distinctively longer	***Pimelodella gracilis***
5	Upper caudal-fin lobe distinctively shorter than the upper lobe; if undamaged, maxillary barbel surpassing the caudal peduncle	***Rhamdia muelleri***
–	Caudal-fin lobes subequal, maxillary barbel not surpassing the caudal peduncle	***Rhamdia quelen***
6	Lower caudal-fin lobe longer than upper lobe	***Phenacorhamdia taphorni***
–	Lower caudal-fin lobe equal or shorter than upper lobe	**7**
7	Lateral-line canal incomplete, extending only to dorsal-fin base	***Nemuroglanis mariai***
–	Lateral-line canal complete, extending to caudal-fin base	**8**
8	Lateral-line canal posteriorly interrupted as a series of short canal segments	***Imparfinis microps***
8	Lateral line continuous	**9**
9	Adipose-fin rectangular	**10**
–	Adipose-fin triangular	**11**
10	Anal-fin origin at vertical through adipose-fin origin, adipose-fin longer than one fourth of SL, six pale bars across dorsum and sides of body	***Chasmocranus rosae***
–	Anal-fin origin anterior to adipose-fin origin, adipose-fin shorter than one fourth of SL, four pale bars across dorsum and sides of body	***Cetopsorhamdia orinoco***
11	First dorsal and pectoral-fin ray extended as a long filament, maxillary barbel surpassing anal-fin base	***Imparfinis pseudonemacheir***
–	First dorsal and pectoral-fin ray not extended as a long filament, maxillary barbel not reaching anal-fin origin	**12**
12	Blotched pattern of body pigmentation	**13**
–	Uniform dark pattern of pigmentation	**14**
13	Pelvic-fin origin approximately at vertical through dorsal-fin origin, adipose-fin base extending far posteriorly beyond distal end of anal-fin	**Undescribed genus and species**
–	Pelvic-fin origin slightly behind mid-length of dorsal-fin base, adipose-fin base ending around same level of distal end of anal fin	***Cetopsorhamdia shermani***
14	Snout conical and conspicuously projected beyond mouth opening, maxillary barbel not surpassing distal end of pectoral fin, dorsal and ventral margins of end of caudal peduncle with a conspicuous white area, caudal fin clear contrasting with dark caudal fin base	**Cetopsorhamdia aff. picklei**
14	Snout depressed, not projected conspicuously beyond mouth opening, maxillary barbel surpassing distal end of pectoral fin, dorsal and ventral margins of end of caudal peduncle dark, pigmented as remaining of body, caudal fin dusky, not contrasting with caudal fin base	***Imparfinis* sp.**
**Pseudopimelodidae**		
1	Lateral line incomplete; premaxillary tooth patch without posterolateral projection; small size	**2**
–	Lateral line complete; premaxillary tooth patch with posterolateral projection; large size	**3**
2	Bar-shaped blotch on caudal-fin base; lateral line reaching a vertical through adipose-fin origin	***Microglanis iheringi***
–	Triangle-shaped blotch on caudal-fin base; lateral line reaching middle of dorsal-fin base	***Microglanis poecilus***
3	Caudal fin lanceolate; pectoral-fin spine covered with thin skin; anterior nares on the margin of the mouth	***Cephalosilurus apurensis***
–	Caudal fin forked; pectoral-fin spine covered with thick skin; anterior nares behind the maxillar barbel insertion	***Pseudopimelodus bufonius***
**Pimelodidae**		
1	Upper jaw projected well beyond lower jaw with premaxillary tooth patch exposed	***Sorubim lima***
–	Lower jaw just slightly or not projected at all, premaxillary tooth plate not completely exposed	**2**
2	Upper portion of caudal-fin base with well-defined dark blotch	**3**
–	Caudal-fin base without well-defined dark blotch	**4**
3	Leading pectoral-fin ray soft and not pungent or spinous	***Megalonema platycephalum***
–	Leading pectoral-fin ray strong, pungent, with anterior and posterior serrae	***Platysilurus mucosus***
4	Eye visible in ventral view, set below midline of head in lateral view; barbels flat and wide	***Hypophthalmus edentatus***
–	Eye not visible in ventral view, set above midline in lateral view; barbels round in cross-section	**5**
5	Body color pattern consisting of dark vertical bars	**6**
–	Body color pattern consisting of dark spots or plain coloration, never with vertical bars	**7**
6	Abundant (more than 50) spots on caudal-fin	***Pseudoplatystoma metaense***
–	Few (less than 50) spots on caudal-fin	***Pseudoplatystoma orinocoense***
7	Body color pattern consisting of numerous spots or blotches, sometimes fussing so that a vermicular pattern appears; interorbital region flat	**8**
–	Body color plain or striped, never with spots or blotches; interorbital region convex	**9**
8	Blotches on body; round vomerine tooth patches separated medially as two independent units	***Leiarius marmoratus***
–	Spots on body; laterally-elongated vomerine tooth patches fused medially as a single wide band, sometimes the medial contact between patches visible	***Zungaro zungaro***
9	Each caudal-fin lobe with dark longitudinal dark stripe from base to tip of caudal fin; distinct blotch on dorsal fin	***Pimelodus ornatus***
–	No dark marks on caudal fin	**10**
10	Giant in adult size; eye small, its diameter much more than three times in dorsal-fin base length; presence of a patch of teeth on roof of mouth	***Brachyplatystoma vaillantii***
–	Small in adult size; eye large, its diameter about three times in dorsal-fin base length, absence of a patch of teeth on roof of mouth	***Pimelodus blochii***
**Auchenipteridae**		
1	Mental barbels absent	**2**
–	Mental barbels present	**3**
2	Caudal fin deeply forked	***Ageneoisus ucayalensis***
–	Caudal fin truncate	***Ageneiosus magoi***
3	7–9 anal-fin rays	***Centromochlus romani***
–	>18 anal-fin rays	**4**
4	Dorsal-fin spine shorter than pectoral-fin spine	***Pseudepapterus hasemani***
–	Dorsal-fin spine longer than or equal to pectoral-fin spine	**5**
5	Caudal fin obliquely truncated	***Trachelyopterus galeatus***
–	Caudal fin forked	***Entomocorus gameroi***
**CHARACIFORMES**		
1	Mouth without teeth	**Curimatidae**
–	Mouth with teeth	**2**
2	Canine teeth present	**3**
–	Canine teeth absent	**6**
3	Adipose fin absent; caudal fin rounded	**Erythrinidae**
–	Adipose fin present; caudal fin not rounded	**4**
4	Dentary canine teeth hypertrophied; caudal fin truncated; rostrum short	**Ctenoluciidae**
–	Dentary without hypertrophied canine teeth; caudal fin bifurcated; rostrum large	**5**
5	Scales ctenoid	**Ctenolucidae**
–	Scales cicloid	**Acestrorhynchidae**
6	Upper jaw without teeth; upper lip with teeth	**7**
–	Upper jaw with teeth; upper lip without teeth	**8**
7	Predorsal process present; mouth evertible	**Prochilodontidae**: ***Prochilodus mariae***
–	Predorsal process absent; mouth non-evertible	**Chilodontidae**
8	Abdominal serrated keel present	**Serrasalmidae**
–	Abdominal serrated keel absent	**9**
9	Branchial membrane fused to isthmus	**Anostomidae**
–	Branchial membrane free from isthmus	**10**
10	Pectoral fins strongly developed and horizontally oriented	**11**
–	Pectoral fins vertically oriented	**12**
11	Fewer than three unbranched rays in pectoral fin	**Parodontidae**
–	Three unbranched rays in pectoral fin	**Crenuchidae**
12	Chest extremely compressed and expanded, forming a ventral keel	**13**
–	Chest not expanded to form ventral keel	**14**
13	Anal-fin origin anterior to dorsal-fin origin; ventral keel well developed	**Gasteropelecidae: *Thoracocharax stellatus***
–	Anal-fin origin posterior to dorsal-fin origin; ventral keel moderately developed	**Triportheidae**
14	Upper lobe of caudal fin longer than lower lobe	**Lebiasinidae**
–	Both lobes of caudal fin of equal size	**15**
15	Two rows of teeth in the dentary	**Bryconidae**
–	One row of teeth in dentary	**16**
16	Anterior margin of maxilla greatly arched above and meeting premaxilla border at right angle	**Iguanodectidae: *Bryconops giacopinni***
–	Anterior margin of maxilla not greatly arched above and not meeting premaxilla border at right angle	**Characidae**
**Curimatidae**		
1	Lateral line incomplete, with only few pored scales	***Curimatopsis* sp.**
–	Lateral line complete with all or almost all lateral scales with pores	**2**
2	Caudal fin with scales reaching two thirds in length in both lobes	***Curimatella immaculata***
–	Caudal fin with scales only on the base of caudal-fin rays	**3**
3	Lateral line with 42 or more scales	**4**
–	Lateral line with 39 or fewer scales	**8**
4	Lateral-line scales more than 90	***Potamorhina altamazonica***
–	Lateral-line scales 42–60	**5**
5	Mouth terminal; dorsal fin without dark spot	**6**
–	Mouth inferior; dorsal fin with or without dark spot	**7**
6	Interorbital width 53–58% of HL; anal-fin rays iii,10-iii,12.; abdominal region, pectoral, pelvic and anal fins with red coloration in live specimens	***Curimata cerasina***
–	Interorbital width less than 52% of HL. Anal-fin rays iii,8-iii,9; abdominal region, pectoral, pelvic and anal fins without red coloration in live specimens	***Psectrogaster ciliata***
7	Dark spot on the dorsal fin; prepelvic region of body not flattened or only obtusely flattened; 1 to 3 scales between posterior border of anus and anal-fin origin	***Steindachnerina pupula***
–	No dark spot on the dorsal fin; prepelvic region of body distinctly flattened; 5 to 7 scales between posterior border of anus and anal-fin origin	***Steindachnerina hypostoma***
8	Without a dark spot on the basal portion of the middle rays of the dorsal fin	***Cyphocharax spilurus***
–	With a dark spot on the basal portion of the middle rays of the dorsal fin	***Steindachnerina argentea***
**Erythrinidae**		
1	Maxilla with 2–3 canine teeth; caudal fin usually spotted; dark lateral stripe absent or diffuse	***Hoplias malabaricus***
–	Maxilla without canine teeth; caudal fin not spotted; dark lateral stripe very well marked	***Hoplerythrinus unitaeniatus***
**Cynodontidae**		
1	Dorsal-fin origin anterior to anal-fin origin	***Hydrolycus armatus***
–	Dorsal-fin origin in line with anal-fin origin	***Raphiodon vulpinus***
**Ctenoluciidae**		
1	In adults, all lateral-line scales perforated (82 or more); body without round spots, except one at the base of caudal fin	***Boulengerella cuvieri***
–	In adults, only up to 25 lateral-line scales perforated; body with black spots	***Boulengerella maculata***
**Acestrorhynchidae**		
1	Lateral line complete with 99–131 scales; dorsal-fin origin anterior to the anal-fin origin	***Acestrorhynchus microlepis***
–	Lateral line incomplete with 33–37 lateral-line scales; dorsal-fin origin posterior to the anal-fin origin	***Gnathocharax steindachneri***
**Chilodontidae**		
1	10 or 11 branched anal-fin rays; anal-fin margin convex or straight; mouth terminal or slightly superior, dorsal fin with a series of dark spots on posterior rays; posterior margin of scales smooth	***Chilodus punctatus***
–	6 to 8 branched anal-fin rays; anal-fin margin somewhat concave in vast majority of specimens; mouth subterminal; dorsal fin with dark pigmentation across distal portions of anterior rays but lacking dark spots on remaining portions of fin; posterior margin of scales somewhat serrate	***Caenotropus labyrinthicus***
**Serrasalmidae**		
1	Adipose-fin base long, its length longer than the distance between posterior margin of dorsal-fin base to the adipose-fin origin	***Metynnis argenteus***
–	Adipose-fin base short, its length shorter than the distance between posterior margin of dorsal-fin base to the adipose-fin origin	**2**
2	Two rows of teeth on the premaxilla	***Mylossoma duriventre***
–	One row of teeth on the premaxilla	**3**
3	Base of dentary teeth circular and separated from adjacent ones; lower jaw prominent and projecting greatly forward from upper jaw	***Catoprion mento***
–	Base of dentary teeth flattened, in contact or very close to adjacent ones; lower jaw equal or slightly anterior to upper jaw	**4**
4	29 or fewer anal-fin rays; snout, head, jaws, and body short and robust; dorsal profile of head to posterior margin of eyes convex; prepelvic and abdominal region red in life; black and conspicuous humeral blotch posterior to opercle	***Pygocentrus cariba***
–	30 or more anal-fin rays; snout, head, jaws, and body long and slender; dorsal profile of head to posterior margin of eyes concave; prepelvic and abdominal region red only during breeding season	**5**
5	Base of posterior premaxillary tooth (6th) approximately equal to adjacent one (5th); ectopterygoid teeth unicuspid, generally 5 or fewer	***Pristobrycon striolatus***
–	Base of posterior premaxillary tooth (6th) wider than adjacent one (5^th^); ectopterygoid teeth bicuspid or tricuspid, generally 7 or more	**6**
6	Posterior margin of caudal fin hyaline, with black vertical bar at base of caudal fin	***Serrasalmus irritans***
–	Caudal fin completely black or with its posterior margin black	**7**
7	Body width 1.7 or less in SL; lateral spots vertically elongated	***Serrasalmus altuvei***
–	Body width 1.7–2.0 in SL; lateral spots rounded	***Serrasalmus rhombeus***
**Anostomidae**		
1	Caudal fin with scales only on its base and without dark bars	**2**
–	Caudal fin with scales at least over two-thirds of both lobes. Caudal-fin with oblique dark bars	***Leporellus vittatus***
2	Dentary teeth tricuspid to pentacuspid; 7 branched anal-fin rays	***Schizodon scotorhabdotus***
–	Dentary teeth incisiform or bicuspid; 8 or more branched anal-fin rays	**3**
3	Body with seven dark vertical bars, the second with a “Y” shape	***Leporinus y-ophorus***
–	Body with rounded spots or longitudinal stripes	**4**
4	Body with 4–5 longitudinal stripes separated by white or yellow stripes	***Leporinus striatus***
–	Body with two rounded black spots at midline	**Leporinus gr. friderici**
**Parodontidae**		
1	Dark vertical bars present; dark lateral stripe absent	***Parodon apolinari***
–	Dark vertical bars absent; dark lateral stripe present	**Parodon aff. suborbitalis**
**Crenuchidae**		
1	Chest naked without scales between pectoral fins	**Characidium gr. boavistae**
–	Chest covered with scales between pectoral fins	**2**
2	Body pigmentation composed by irregular lines and dots	***Characidium pteroides***
–	Body pigmentation composed by well-defined vertical bars	**3**
3	9 or fewer branched pectoral-fin rays	4
–	10 or more branched pectoral-fin rays	**5**
4	All vertical bars always originating on dorsum; body depth 23% of HL or less	**Characidium gr. zebra**
–	Not all vertical bars originating on dorsum, thin vertical bars not reaching the dorsum between vertical bars originating on dorsum; body depth 24% of HL or more	***Characidium* sp.**
5	Mid-lateral stripe diffuse or absent; vertical bars fragmented in rhomboidal shape	**Characidium cf. steindachneri**
–	Mid-lateral stripe very well developed; vertical bars continuous and with dots appearance at junction with mid-lateral line	***Characidium chupa***
**Triportheidae**		
1	Pectoral fin not surpassing ventral-fin origin	***Engraulisoma taeniatum***
–	Pectoral fin surpassing ventral-fin origin	**2**
2	24–27 branched anal-fin rays; 5–6 scale rows between lateral line and dorsal-fin origin	***Triportheus venezuelensis***
2	28–32 branched anal-fin rays; 7 scale rows between lateral line and dorsal-fin origin	***Triportheus orinocensis***
**Lebiasinidae**		
1	Adipose fin present	***Lebiasina erythrinoides***
–	Adipose fin absent	**2**
2	One row of teeth in the premaxilla; maxilla S curved; dark lateral stripe complete and well-marked (from mouth to caudal fin)	***Copella arnoldi***
–	Two rows of teeth on the premaxilla; maxilla not S curved; dark lateral stripe incomplete and thin (from mouth to few scales after head)	***Pyrrhulina lugubris***
**Bryconidae**		
1	Two rows of teeth in the premaxilla	***Salminus* sp.**
–	Three rows of teeth in the premaxilla	**2**
2	Dark lateral stripe present, from opercle to tip of middle caudal-fin rays; upper caudal-fin lobe unpigmented	***Brycon whitei***
–	Dark lateral stripe absent; upper caudal-fin lobe pigmented	***Brycon falcatus***
**Characidae**		
1	Lateral line incomplete	**2**
–	Lateral line complete	**16**
2	Dorsal fin with a black blotch	**3**
–	Dorsal fin without blotches	**4**
3	Body height 26.6 to 29.3% of SL	***Hyphessobrycon dorsalis***
–	Body height is 34.5 to 36.9% of SL	***Hyphessobrycon sweglesi***
4	One row of premaxillary teeth	**5**
–	Two rows of premaxillary teeth	**7**
5	Dorsal-fin origin posterior to the anal-fin origin	***Paragoniates alburnus***
–	Dorsal-fin origin anterior to the anal-fin origin	**6**
6	A dark embedded crescent-shaped mark on base of each caudal lobe; no red color on caudal fin	***Microschemobrycon casiquiare***
–	Without such dark pigmentation on the base of caudal fin; caudal fin red in life	***Aphyocharax alburnus***
7	Caudal fin with black pigmentation in both lobes	***Hyphessobrycon otrynus***
–	Caudal fin without black pigmentation	**8**
8	Anal-fin base with a conspicuous dark band	***Hemigrammus barrigonae***
–	Anal-fin base without dark pigmentation	**9**
9	25–27 branched anal-fin rays; caudal peduncle red or rose in life	***Hemigrammus stictus***
–	14–22 branched anal-fin rays; no red or pink color on caudal peduncle	**10**
10	A small dark line of black chromatophores along the anal- and caudal-fin bases	***Cyanogaster noctivaga***
–	Caudal fin without dark coloration or with a well-developed spot at its base or on caudal-fin lobes	**11**
11	With a conspicuous caudal spot	**12**
–	Without a caudal spot or with a lateral stripe continuing along middle caudal-fin rays, but not forming a caudal spot	**14**
12	Dark melanophores present on sides of body and between anal fin and lateral line	***Hyphessobrycon metae***
–	No dark melanophores present on sides of body and between anal fin and lateral line	**13**
13	Caudal spot triangle-shaped covering the central portion of caudal-fin base, continuing along middle caudal-fin rays and usually not in contact with the lateral stripe	***Hemigrammus micropterus***
–	A large caudal spot covering entire caudal-fin base, continuing along middle caudal-fin rays and in contact with lateral stripe	***Hemigrammus newboldi***
14	Body depth 27% or less of SL; 14–16 branched anal-fin rays; maxillary teeth absent; black blotch on upper caudal-fin lobe	***Hyphessobrycon diancistrus***
–	Body depth 28% or more of SL; 18–20 branched anal-fin rays; maxillary teeth present; without dark blotch on upper caudal-fin lobe	**15**
15	12 dentary teeth; 6–7 perforated lateral-line scales. Without a dark blotch at caudal peduncle	***Hyphessobrycon taguae***
–	14 dentary teeth; 9–10 perforated lateral-line scales. With a dark blotch at caudal peduncle	***Hyphessobrycon acaciae***
16	Dorsal-fin origin at or posterior to vertical through anal-fin origin	**17**
–	Dorsal-fin origin anterior to the anal-fin origin	**22**
17	61–68 anal-fin rays	***Xenagoniates bondi***
–	25–55 anal-fin rays	**18**
18	Length of maxilla equal or shorter than vertical diameter of eye; adult males with hypertrophied caudal-fin squamation on lower caudal-fin lobe	***Gephyrocharax valencia***
–	Length of maxilla longer than vertical diameter of eye; adult males without hypertrophied caudal-fin squamation	**19**
19	No external mammiliform teeth	***Charax* sp.**
–	External mammiliform teeth present in the maxilla and premaxilla	**20**
20	50–65 lateral-line scales	***Roeboides dientonito***
–	66 or more lateral-line scales	**21**
21	66–70 perforated lateral-line scales; 12–14 gill rakers on lower arm of the first gill arch	***Roeboides* sp.**
21	70–88 perforated lateral-line scales; 7–11 gill rakers on lower arm of the first gill arch	***Roeboides affinis***
22	16 or less total anal-fin rays	**23**
–	17 or more total anal-fin rays	**27**
23	Premaxillary teeth arranged in 2 rows; Anterior triad of larger teeth with rounded base absent	***Ceratobranchia* sp.**
–	Premaxillary teeth arranged in 3 rows; Anterior triad of larger teeth with rounded base present	**24**
24	Dorsal-fin origin anterior to pelvic-fin insertion	***Creagrutus* sp.**
–	Dorsal-fin origin posterior or aligned with pelvic-fin origin	**25**
25	Third infraorbital poorly developed, leaving a broad gap between its posterior margin and the horizontal limb of preopercle	***Creagrutus bolivari***
–	Third infraorbital well-developed contacting or with a small gap from horizontal limb of preopercle	**26**
26	38–42 lateral-line scales; interorbital distance 34.0–37.9% of HL	***Creagrutus atratus***
–	36–38 lateral-line scales; interorbital distance 28.6–35.4% of HL	**Creagrutus cf. taphorni**
27	40 or more total anal-fin rays	**28**
–	39 or less total anal-fin rays	**33**
28	Cycloid scales on body sides	**29**
–	Ctenoid scales on body sides, or at least in preventral area	**30**
29	Maxilla shorter than vertical diameter of eye; cleithrum without a notch on posteroventral portion; body sides with dark sinuous lines	***Markiana geayi***
–	Maxilla longer than vertical diameter of eye; cleithrum with notch on posteroventral portion, near base of posteriorly directed spiniform projection; body sides with dark sinuous lines	***Charax metae***
30	58 or fewer lateral-line scales; premaxillary and dentary teeth multicuspid; ctenoid scales only on preventral area	***Ctenobrycon spilurus***
–	59 or more lateral-line scales; one or more conical or canine teeth; ctenoid scales on entire body	**31**
31	100–110 lateral-line scales	***Cynopotamus bipunctatus***
–	70–84 lateral-line scales	**32**
32	35–38 branched anal-fin rays; 73–76 lateral-line scales	***Acestrocephalus sardina***
–	39–45 branched anal-fin rays; 79–84 lateral-lines scales	***Galeocharax gulo***
33	A pair of cuspidate teeth on premaxilla pointing forward on labial sides of upper jaw; two large black blotches on body sides, one anterior to dorsal-fin origin and another on caudal peduncle	***Exodon paradoxus***
–	No cuspidate teeth on premaxilla; different color pattern than that described above	**34**
34	One row of premaxillary teeth	**35**
–	Two rows of premaxillary teeth	**38**
35	Premaxillary teeth with five cusps, the outer cusps very small; dentary teeth with five cusps, the three central cusps flat and approximately equal in size and the outer cusps very small	***Cheirodontops geayi***
–	Premaxillary teeth with nine cusps; dentary teeth heptacuspid, with central cusp longer than others	**36**
36	A remarkable elongation of the second unbranched dorsal-fin ray in males; maxilla somewhat triangular, short, with mid-length portion deeper and gradually narrowing to the posterior tip	***Odontostilbe pao***
–	No elongation of the second unbranched dorsal-fin ray in males; maxilla somewhat spatula-like shaped, short or elongate, with a deep mid-length region, narrowing abruptly at posterior tip	**37**
37	Adipose-fin origin at vertical through second or third last anal-fin ray insertion; mature males with hooks on first to sixth-seventh branched anal-fin rays	***Odontostilbe splendida***
–	Adipose-fin origin at vertical through last anal-fin ray insertion; mature males with hooks on first to twenty second branched anal-fin rays	***Odontostilbe pulchra***
38	Presence of a predorsal spine; anterior three anal-fin rays black	***Poptella compressa***
–	No predorsal spine; three anterior anal-fin rays unpigmented	**39**
39	33–38 total anal-fin rays	**40**
–	32 or less total anal-fin rays	**42**
40	Body depth 28.0–37.0% of SL; maxilla with 20–30 conical teeth; 6 scale rows between lateral line and dorsal-fin origin; one humeral spot	***Phenacogaster maculoblonga***
–	Body depth 38.0% or more of SL; maxilla with 1–3 multicuspidate teeth; 7–9 scale rows between lateral line and dorsal-fin origin; two humeral spots	**41**
41	Caudal peduncle with a large dark spot	***Tetragonopterus argenteus***
–	Caudal peduncle without spot	***Gymnocorymbus bondi***
42	16–20 total anal-fin rays	**43**
–	23–32 total anal-fin rays	**44**
43	17–18 anal-fin rays	***Knodus deuterodonoides***
–	16 anal-fin rays	**Bryconamericus cf. cismontanus**
44	Spine-like pelvic bones projecting anteriorly from pelvic-fin base, with distal tip free from musculature	***Jupiaba polylepis***
–	No spine-like pelvic bones	**45**
45	4 teeth on the inner row of premaxilla	**46**
–	5 teeth on the inner row of premaxilla	**48**
46	3 or fewer teeth on maxilla	***Knodus alpha***
–	More than three teeth on maxilla	**47**
47	5 or fewer maxillary teeth in adult individuals (>5 cm SL); second premaxillary tooth of inner row pentacuspid; 12–13 predorsal scales; fins of live specimens reddish	***Hemibrycon loisae***
–	6 or more maxillary teeth in adult individuals (>5 cm SL); second premaxillary tooth of inner row heptacuspid; 14–16 predorsal scales; fins of live specimens not reddish	***Hemibrycon metae***
48	One or both caudal-fin lobes with dark coloration	**49**
–	Both caudal-fin lobes without distinct pigmentation	**50**
49	Only upper caudal-fin lobe with dark pigmentation	***Moenkhausia lepidura***
–	Both caudal-fin lobes with black pigmentation	***Moenkhausia dichoura***
50	Middle caudal-fin rays without dark coloration	***Moenkahusia copei***
–	Middle caudal-fin rays with dark coloration	**51**
51	Predorsal line naked	***Astyanax bimaculatus***
–	Predorsal line with scales	**52**
52	Base of anal fin with an oblique dark stripe extending across caudal peduncle and onto middle and upper caudal-fin rays	***Astyanax metae***
–	Base of anal fin without oblique dark stripe	**53**
53	45 or more lateral-line scales	***Astyanax integer***
–	Fewer than 45 lateral-line-scales	***Astyanax venezuelae***
**Cichlidae**		
1	African lips (posterior portion of the lower lip not covering part of the upper lip); three roundish ocellar blotches in adults (large and oval blotches in juveniles)	***Cichla orinocensis***
–	American lips (posterior portion of the lower lip covering part of the upper lip); different color pattern than above	**2**
2	Bone expansion in the upper region of the first gill arch forming a well-developed fleshy lobe	**3**
–	First gill arch without such a lobe	**6**
3	Lower pharyngeal bone without teeth along its margin	***Mikrogeophagus ramirezi***
–	Lower pharyngeal bone with teeth along its margin	**4**
4	Lateral spots on body present; two pectoral spots	***Apistogramma macmasteri***
–	No lateral spots on body; with or without a single pectoral spot	**5**
5	Pectoral fin base with spot; with distinct abdominal stripes	***Apistogramma hongsloi***
–	Without spot on pectoral fin base; without abdominal stripes	***Apistogramma hoignei***
6	Irregular predorsal scale pattern	**7**
–	Uniserial or triserial predorsal scale pattern	**11**
7	Six or seven anal-fin spines; 24–30 scales in upper lateral line series; vertical bars 6 and 7 parallel; body deep (50.7–55.6%of SL)	***Mesonauta egregius***
–	Three anal-fin spines; 40 or more scales in upper lateral line series; elongate and somewhat cylindrical body (less than 50.0% of SL)	**8**
8	Humeral blotch present	**9**
–	Humeral blotch absent	**11**
9	Lateral line crossing middle portion of humeral blotch	***Crenicichla alta***
–	Lateral line crossing upper portion of humeral blotch	**10**
10	Chain of blotches along lateral lines; length of posterior dorsal-fin spine 9.6–10.6% of SL; caudal peduncle length at ventral part 10.0–11.5% of SL; length of ventral fin 19.4–20.4% of SL	***Crenicichla sveni***
–	No chain of blotches along lateral lines; length of posterior dorsal-fin spine 10.8–11.2% of SL; caudal peduncle length at ventral part 11.8–12.2% of SL; length of ventral fin 17.3–18.2% of SL	***Crenicichla* sp.**
11	Maxilla extending posterior to the anterior margin of eye; caudal-fin length (from caudal-fin base to tips of middle caudal-fin rays) 16.2–24.6% of SL; head depth at orbit level 16.7–21.6% SL; caudal peduncle depth where least 10.7–14.2% of SL	***Crenicichla geayi***
–	Maxilla only reaching anterior margin of eye; caudal-fin length (from caudal-fin base to tips of middle caudal-fin rays) 22.7–24.7% of SL; head depth at orbit level 15.1–15.6% SL; caudal peduncle depth where least 9.34–9.83% of SL	**Crenicichla gr. wallacii**
12	Uniserial predorsal scale pattern	**13**
–	Triserial predorsal scale pattern	**14**
13	8 branched anal-fin rays; caudal fin rounded; less than 8 scales in the lower lateral line	***Andinoacara* sp.**
–	7 branched anal-fin rays; caudal fin subtruncate-truncate; more than 8 scales in the lower lateral line	***Bujurquina mariae***
14	Dark stripe extending from posterodorsal margin of eye to lower angle of preopercle	***Aequidens metae***
–	Dark stripe restricted to a suborbital blotch only	**14**
15	Head sides with bluish or greenish iridescent stripes (in live specimens)	***Aequidens diadema***
–	Head sides without bluish or greenish iridescent stripes (in live specimens)	***Aequidens tetramerus***
**Sciaenidae**		
1	Lateral-line scales not covered by smaller scales; body depth 3.8–4.3% of SL; predorsal distance 3.0–3.1% of SL; anal-fin base 5.9–9.3% of SL; caudal peduncle length 3.4–3.7% of SL; postorbital length 2.0–2.4% of SL; length of second anal-fin spine 1.8–2.3 % of HL	***Pachyurus gabrielensis***
–	Lateral-line scales covered by smaller scales; body depth 3.0–3.8% of SL; predorsal distance 2.7–3.0% of SL; anal-fin base 11.2–17.1% of SL; caudal peduncle length 3.8–4.7% of SL; postorbital length 1.6–2.0% of SL; length of second anal-fin spine 2.6–6.0% of HL	***Plagioscion squamosissimus***

## Discussion

Regional checklists of freshwaters fishes become dynamic over time as studies in freshwater fish taxonomy and distribution advance for the Neotropical region (Reis et al. 2016). The most recent checklist for the Meta River basin reported 258 species for the Cusiana River sub-basin (Usma-Oviedo et al. 2016); it is a higher number of species than reported herein because re-identification and taxonomic updating processes of specimens excluded 59 of the 258 nominal species reported by Usma-Oviedo et al. (2016). For example, most of the undetermined (e.g., *Ancistrus* sp., *Aphyocharax* sp, *Microglanis* sp., and *Odontostilbe* sp.) and erroneous records (e.g., *Hemibrycon cristiani*, *Pyrrhulina brevis*, *Schultzichthys gracilis*, and *Steindachnerina guentheri*) originally counted as independent species were merged with other recorded species after our verification of the data. This is not surprising, taxonomy proceeds at a faster pace than the institutional ability to maintain updated records. For this reason, there is a need to account for validation of species identification when regional checklists are assembled from multiple secondary sources in order to avoid errors due to outdated or unverified data.

Extrapolation suggests that the drainage could have a richness of roughly 314 species, indicating that the number of species found in the present study represents 77.7% of the expected richness in the area (Table 1). However, this estimate represents a rough estimate because sampling efforts have not been uniform across the drainage. A historical sampling-specific bias is not expected in the Cusiana River sub-basin, with the possible exception of an elevational bias (as is true for the whole Orinoco drainage in Colombia). Given the non-uniform nature of sampling in comparable river systems, we suggest that our extrapolations of species richness may be useful for comparisons among drainages using collection records as the input for rough estimation. This is particularly important since most checklist studies compare observed and not estimated richness, with the latter a more appropriate measure because it incorporates (and even overestimates) uncertainty from the samples into the estimation, and it also serves accounts for sampling effort among drainages.

Among similar Orinoco Andean tributaries, the Cusiana is one of the best-sampled sub-basins, exceeding in species richness other recently well-sampled sub-basins such as Orotoy (113 spp.; Ramírez-Gil et al. 2011) and Pauto (182 spp.; Maldonado-Ocampo et al. 2013). In fact, the Cusiana River sub-basin represents the quantile 0.83 among Orinocoan drainages, indicating that 83 % of the other drainages had fewer than 74 localities represented in the collections. The importance of the Cusiana River sub-basin is not only determined by its fish richness, but also because of its diverse and extensive aquatic ecosystem richness (rivers, streams, lagoons, estuaries, palm swamps, riparian forests, and flooded savannas) that provide important areas for fish reproduction, shelter and food. Because aquatic ecosystems have dynamic ecological and environmental processes (Teresa et al. 2015, Ribeiro et al. 2016, Toussaint et al. 2016), management and conservation projects of sub-basins should be addressed at regional (sub-basin) scales.

The documentation of the ichthyofauna in cis-Andean Colombian sub-basins has been increasing during the last decade, but new records and species can likely still be found in areas previously thought to be well-sampled (e.g., Ballen 2011, Vanegas-Ríos et al. 2015, Ballen et al. 2016a, 2016b, Burns et al. 2017, García-Alzate et al. 2017). Most of the sampling effort has been carried out in the piedmont and lowland areas in the Cusiana as well as in other sub-basins, and exploration of High Andean areas could lead to the discovery of local endemic species at the basin scale that usually are underestimated (Carvajal-Quintero et al. 2015).

Sub-basins adjacent to the Cusiana draining along the eastern slope of the Eastern Cordillera in the Orinoco region of Colombia (e.g., Guachiría, Casanare, Upía, Túa, and Cravo Sur) have not been well sampled and their richness is surely underestimated (Urbano-Bonilla et al. 2014). Continuous efforts are still to be carried out in order to document the fish fauna present along this region; this information is crucial to better understand how different anthropogenic activities (mining, oil extraction, agricultural, and livestock practices) are affecting the environmental conditions of these areas and as a consequence, the fish that live therein. Combination of this kind of information and further environmental data is a necessary step in order to generate freshwater conservation strategies using different approaches and therefore go further toward effective protection initiatives for species subject of conservation in the region.
